# Influence of delta-doping on the hole capture probability in Ge/Si quantum dot mid-infrared photodetectors

**DOI:** 10.1186/1556-276X-9-504

**Published:** 2014-09-16

**Authors:** Andrew Yakimov, Victor Kirienko, Vyacheslav Timofeev, Aleksei Bloshkin, Anatolii Dvurechenskii

**Affiliations:** 1 Rzhanov Institute of Semiconductor Physics, Siberian Branch of the Russian Academy of Science, 630090 Novosibirsk, Russia; 2 Tomsk State University, 634050 Tomsk, Russia

**Keywords:** Quantum dots, Silicon, Germanium, Interband transitions, Infrared photodetectors

## Abstract

We study the effect of delta-doping on the hole capture probability in ten-period *p*-type Ge quantum dot photodetectors. The boron concentration in the delta-doping layers is varied by either passivation of a sample in a hydrogen plasma or by direct doping during the molecular beam epitaxy. The devices with a lower doping density is found to exhibit a lower capture probability and a higher photoconductive gain. The most pronounced change in the trapping characteristics upon doping is observed at a negative bias polarity when the photoexcited holes move toward the *δ*-doping plane. The latter result implies that the *δ*-doping layers are directly involved in the processes of hole capture by the quantum dots.

## Background

In the past several years, there has been a surge of interest in nanostructures that exhibit quantum confinement in three dimensions, known as quantum dots (QDs). The potential advantages of the quantum dot infrared (IR) photodetectors (QDIPs) as compared with two-dimensional systems are as follows [1,2]: (i) an increased sensitivity to normally incident radiation as a result of breaking of the polarization selection rules, so eliminating the need for reflectors, gratings, or optocouplers; (ii) an expected large photoconductive gain associated with a reduced capture probability of photoexcited carriers due to suppression of electron-phonon scattering; and (iii) a small thermal generation rate, resulted from a zero-dimensional character of the electronic spectrum that renders a much improved signal-to-noise ratio. The operation of the QDIP as a photodetector is associated with the escape of electrons or holes from QD stimulated by the absorption of the IR photons. Most of the demonstrations of QDIPs were achieved with *n*-type III to V self-assembled nanoheterostuctures. Only limited studies of *p*-type QDIPs have been reported [3,4]. The attractive features of *p*-QDIPs include a well-preserved spectral profile [3], as an opposite to a conventional *n*-type response strongly dependent on applied bias, increased density of states, and lower dark current due to the higher hole effective mass [4].

SiGe-based QDIPs represent another attractive type of the device due to its compatibility with standard Si readout circuitry. At present, the most highly developed technology for fabricating arrays of SiGe-based QDs utilizes strain-driven epitaxy of Ge nanoclusters on Si(001) surface [5]. The photoresponse of *p*-type Ge/Si heterostructures with QDs in the mid-wave atmospheric window was observed by several groups [6-12] and attributed to the transitions from the hole states bound in Ge QDs to the continuum states of the Si matrix.

One figure of merit that determines the photoconductive gain and hence the detector responsivity and detectivity is the probability that a carrier is captured by a QD after its optical generation. In particular, a background-limited detectivity increases with the increase of capture probability *p*, while in a dark current limited condition, the detectivity falls with the increase of *p*[13]. The understanding of the carrier trapping mechanism associated with the carrier transport behavior is an important issue for optimizing the detectivity of QDIPs. Most of the reported QDIPs incorporate delta-doping barrier regions [9,10,12,14,15] instead of directly doped QD layers [7,10,16]. Placing dopants away from the dot layers generally increases the photoresponse due to the appearance of a built-in electric field [12] and a reduced number of point defects in the active QD region [15]. The carrier capture rate into the QDs depends on the distribution of the electric field in some areas surrounding QDs. In order to achieve an efficient carrier transfer, the doping plane lies only a few nanometers beyond the dot layer and thereby should strongly affect the carrier capture probability. In this paper, we present a study of influence of boron delta-doping on the hole capture probability of Ge/Si QDIPs.

## Methods

**Figure 1:**
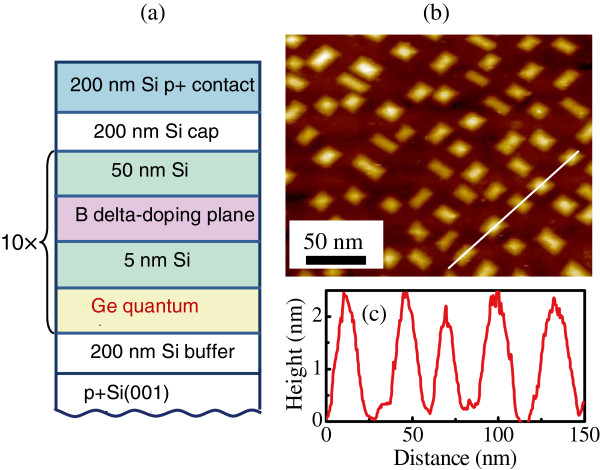
**Device structure of the Ge/Si QDIP.(a)** Layer sequence in the Ge/Si quantum dot photodetector. **(b)** STM image from topmost uncapped Ge layer. **(c)** The STM cross-sectional height profile along the white line.

Figure 1a shows schematically the structure of the detector discussed in this paper. The samples were grown by solid source molecular beam epitaxy on a (001) oriented boron-doped *p*^+^-Si substrate with resistivity of 0.05 *Ω* cm. The active region of the device was composed of ten stacks of Ge quantum dots separated by 55-nm Si barriers. Each Ge QD layer consisted of a nominal Ge thickness of about 6 monolayers (ML) and formed by self-assembling in the Stranski-Krastanov growth mode at 500°C and at a growth rate of 0.2 ML/s for all samples. From scanning tunneling microscopy (STM) experiments with uncapped samples, we observed the Ge dots to be approximately 15 to 20 nm in lateral size (Figure 1b) and about 1.5 to 2.0 nm in height (Figure 1c). They have the form of hut clusters bounded by {105} facets. The density of the dots is about (1−2)×10^11^ cm ^−2^. The Si barriers were deposited at 600°C.

The active region was sandwiched in between the 200-nm-thick intrinsic Si buffer and cap layers. Finally, a 200-nm-thick *p*^+^-Si top contact layer (5×10^18^ cm ^−3^) was deposited. The *p*-type remote doping of the dots was achieved with a boron *δ*-doping layer inserted 5 nm above each dot layer. The areal doping density was *N*_B_=2×10^11^ cm ^−2^. For vertical photocurrent measurements, the samples were processed in the form of circular mesas with a diameter of 3 mm by using wet chemical etching and contacted by Al:Si metallization. The bottom contact is defined as the ground when applying voltage to the detector.

To trace the effect of doping on the hole capture process of the same sample, we used the hydrogen passivation technique. Hydrogen is commonly used in Si-based photovoltaic cells to neutralize shallow acceptor [17,18] and donor [19] impurities, to passivate deep recombination [20] and nonradiative centers [21]. In our experiments, atomic hydrogen was introduced into the samples by a treatment from a remote radio-frequency plasma at a substrate temperature of 300°C for 15 and 30 min. The passivation system consists of a quartz tube (diameter of 1 cm) with ring-like electrodes through which H _2_ flowed at 200 cm ^3^/min at a pressure of approximately 1 mbar. Radio frequency radiation (40.7 MHz, 70 W) generated the plasma inside the tube, and the sample was located 8 cm downstream from the tube nozzle on a heater block.

The normal-incidence photoresponse was obtained using a Bruker Vertex 70 Fourier transform IR spectrometer (Bruker Optik Gmbh, Ettlingen, Germany) with a spectral resolution of 5 cm ^−1^ along with a SR570 low-noise current preamplifier (Stanford Research Systems, Sunnyvale, CA, USA). The temperature for all measurements is 80 K. The photocurrent (PC) spectra were calibrated with a DLaTGS detector. The noise characteristics were measured with an SR770 fast Fourier transform analyzer (Stanford Research Systems), and the white noise region of the spectra was used to determine the detectivity. The sample noise was obtained by subtracting the preamplifier-limited noise level from the experimental data. The dark current was tested as a function of bias by a Keithley 6430 Sub-Femtoamp Remote SourceMeter (Keithley Instruments Inc., Cleveland, OH, USA). The devices were mounted in a cold finger inside a Specac cryostat with ZnSe windows. For dark current and noise measurements, the samples were surrounded with a cold shield.

## Results and discussion

**Figure 2:**
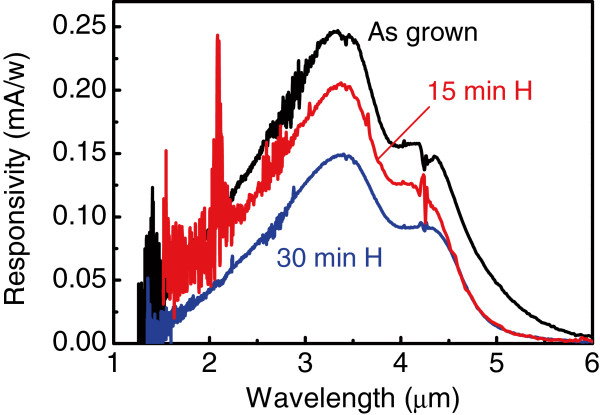
**Responsivity spectra measured for the as-grown and the hydrogenated samples.** The data were taken in a photovoltaic regime when no bias voltage is applied. The hydrogen plasma exposure duration was 15 and 30 min.

Figure 2 depicts the mid-infrared PC spectra measured at zero applied voltage in the as-grown and hydrogen passivated samples. As we have demonstrated earlier [12], the photovoltaic dual-peak spectral response centered around 3.4 μm is a direct consequence of a built-in electric field caused by charge redistribution between QDs and *δ*-doping layers and originates from the hole intraband transitions. The decrease in peak responsivity after hydrogenation is consistent with the reduced hole density in the dots due to neutralization of boron dopants in the *δ*-doping layers.

From noise measurements, we established that the noise level in the photoconductive mode is dominated by a generation-recombination noise. In this case, when the carrier capture probability into a QD is small, the photoconductive gain can be expressed as

(1) $$g=i_{n}^{2}/\left(4eI_{d}\mathrm{\Delta f}\right),$$

where *e* is the charge of an electron, *i*_*n*_ is the noise current, *I*_*d*_ is the dark current, and *Δf* is the noise bandwidth. The capture probability *p* is related to the gain through [22]

(2) $$g=\frac{1-p/2}{\text{FNp}},$$

where *F* is the fill factor which describes the area coverage of the QDs in a dot layer and *N* is the number of QD layers. The fill factor of ≈0.32 was estimated from STM data presented in Figure 1b. For the evaluation of the gain, we have subtracted the thermal (Johnson) noise from the measured noise in order to have the pure generation-recombination noise. The Johnson noise was calculated as $i_{J}=\sqrt{4\mathrm{kT\Delta f}/\rho }$, where *k* is the Boltzmann’s constant, *T* is the temperature, and *ρ* is the differential resistance, which is extracted from the dark current measurements.

**Figure 3:**
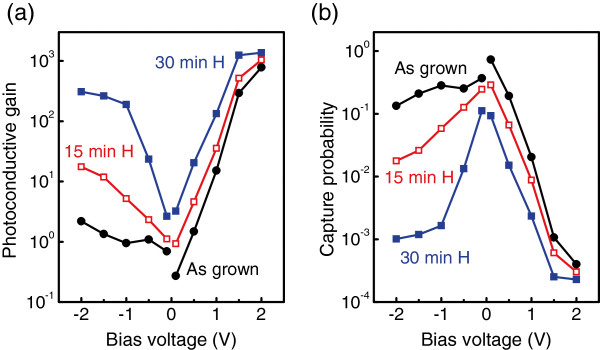
**Photoconductive gain and hole capture probability.** Photoconductive gain and hole capture probability of the as-grown and hydrogenated samples. **(a)** Photoconductive gain as a function of applied bias. The gain was found using noise current and dark current data measured in the experiment. **(b)** Hole capture probability vs bias calculated by using Equation 2 with *F*=0.32, *N*=10. The plasma exposure duration was 15 and 30 min.

The gain and hole capture probability calculated using Equations 1 and 2 are shown in Figure 3. For the as-grown samples, the gain is less than unity (*p*≈1) at low bias values, while it increases above 0.5 V as a result of reduced capture probability. At 2 V, *g*=800 and *p*=4×10^−4^. Note that quantum well IR photodetectors exhibit gains in the range from 0.1 to 1 [23]. The higher gain in QDIPs is generally attributed to the phonon bottleneck effect [24] suppressing the carrier capture accompanied by the optical phonon emission. In the hydrogenated samples, the gain is found to be much higher than unity for all applied biases and reaches the value of 1,400 at approximately 2 V.

**Figure 4:**
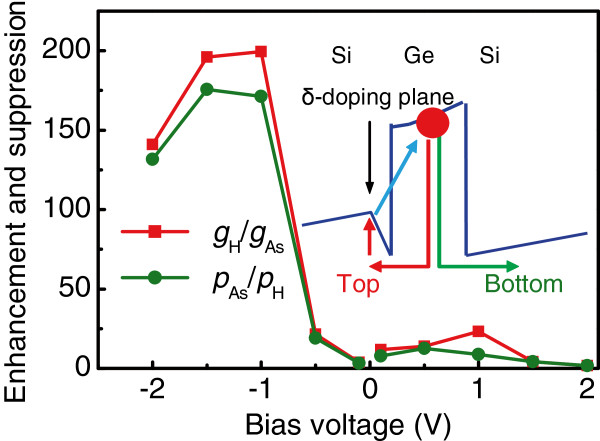
**Enhancement of the gain and suppression of the hole capture probability by hydrogen passivation.** The gain enhancement factor and the suppression factor for carrier capture probability are determined as *g*_H_/*g*_As_ and *p*_As_/*p*_H_, where *g*_As_, *p*_As_, *g*_H_, and *p*_H_ are the gain and capture probability before and after exposure to a hydrogen plasma for 30 min, respectively. Inset shows a valence band diagram with the involved hole transitions in the QDIP.

Figure 4 illustrates the effect of hydrogen plasma on the enhancement of the gain and on the suppression of the hole capture. Here we determine the gain enhancement factor as *g*_H_/*g*_As_ and the suppression factor for carrier trapping probability as *p*_As_/*p*_H_, where *g*_As_, *p*_As_, *g*_H_, and *p*_H_ are the gain and capture probability before and after exposure to a hydrogen plasma for 30 min, respectively. A considerable suppression of the hole capture process and enhancement in the gain by hydrogen passivation are observed at a negative bias polarity when the holes move toward the nearest doping plane. The maximum suppression factor is about 170 at −1.5 V. This result undoubtedly points out that the presence of the *δ*-doping plane near the QD layer yields more efficient hole trapping in QDs. The probable explanation for a single QD layer is based on a four-zone trapping mechanism proposed for quantum well intersubband photodetectors in [25] and is illustrated in an inset of Figure 4. At a positive bias polarity, the photoexcited holes drift toward the bottom electrode without any trapping (green arrow) thus giving rise to a large photoconductive gain. When the negative voltage is applied, the emitted holes can be captured by the negatively charged boron impurities of the nearest *δ*-doping layer (red arrows) and finally populate the adjacent QD by crossing the tunneling region of the Si layer above the dots (blue arrow). Neutralization of dopants by hydrogen passivation suppresses this mechanism of hole trapping and results in a decrease of carrier capture probability.

**Figure 5:**
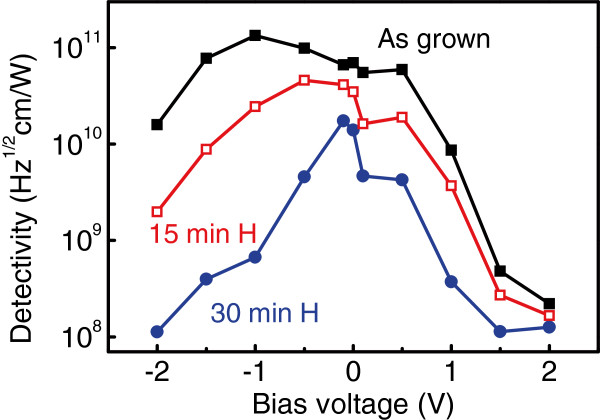
**Detectivity measured for the as-grown and the hydrogenated samples.** As a function of applied bias. The plasma exposure duration was 15 and 30 min.

The specific detectivity is given by $D^{⋆}=R\sqrt{A\cdot \mathrm{\Delta f}}/i_{n}$, where *R* is the responsivity and *A* is the device area. The detectivity obtained from the devices is shown in Figure 5. One can see that hydrogenation reduces the detectivity as well as capture probability. The reduction of *D*^⋆^ with the decrease of *p* is a signature of a background-limited performance [13], which was really observed in our devices at *T*<110 K [12,26].

**Figure 6:**
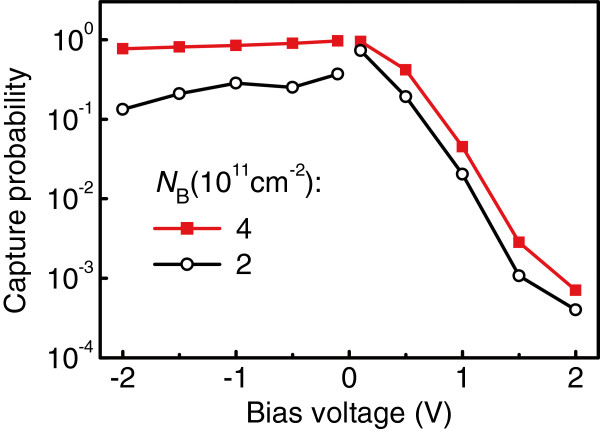
**Hole capture probability.** As a function of applied bias for Ge/Si QDIPs in which the sheet boron density in the *δ*-doping layers *N*_B_ was 4×10^11^ and 2×10^11^ cm ^−2^.

To demonstrate the effect of delta-doping on the hole capture rate once more, we fabricated another sample under conditions similar to those described above, except that a sheet boron concentration in each doping layer was increased by a factor of two (i.e., *N*_B_=4×10^11^ cm ^−2^). This QDIP was not treated with the hydrogen plasma. One can see in Figure 6 that an increase of doping density leads to a higher capture probability. Again, the most pronounced change in *p* occurs at a negative bias. This is definitely in agreement with the hydrogen plasma experiments.

## Conclusions

The photoconductive gain and the hole capture probability of a *δ*-doped mid-infrared Ge quantum dot photodetector subjected to the hydrogen plasma exposure was investigated. The devices after the plasma treatment show a significant increase of the gain and a reduction of the capture rate most likely due to the neutralization of impurity charge in the *δ*-doping layers. An increase of a doping density in the as-grown devices was found to yield a higher capture probability and a lower gain. The change in the device characteristics with the change of the doping density turns out to be most pronounced when the photoexcited holes move toward the doping layers. These results indicate that placing dopants in the barriers has a great effect on the probability that a hole is trapped on a Ge QD.

## Abbreviations

IR: infrared; ML: monolayer; PC: photocurrent; QD: quantum dot; QDIP: quantum dot infrared photodetector; STM: scanning tunneling microscopy.

## Competing interests

The authors declare that they have no competing interests.

## Authors’ contributions

AY conceived and designed the experiment, carried out the photocurrent measurements, participated in the coordination, and drafted the manuscript. VK and VT prepared the samples using a molecular beam epitaxy technique and hydrogen plasma treatment. AB performed the noise measurements. AD supervised the project work. All authors read and approved the final manuscript.
